# Clinical implications of morular metaplasia in fertility-preserving treatment for atypical endometrial hyperplasia and early endometrial carcinoma patients

**DOI:** 10.1007/s00404-021-06382-3

**Published:** 2022-03-04

**Authors:** Pengfei Wu, Qiaoying Lv, Jun Guan, Weiwei Shan, Xiaojun Chen, Qin Zhu, Xuezhen Luo

**Affiliations:** 1 Department of Gynecology, Obstetrics and Gynecology Hospital, Fudan University, No. 419, Fangxie Road, Shanghai, 200011 People’s Republic of China; 2 Shanghai Key Laboratory of Female Reproductive Endocrine Related Diseases, Shanghai, 200011 People’s Republic of China; 3 Department of Pathology, Obstetrics and Gynecology Hospital, Fudan University, Shanghai, 200011 People’s Republic of China

**Keywords:** Endometrial metaplasia, Morular metaplasia, Atypical endometrial hyperplasia, Endometrioid carcinoma, Conservative treatment

## Abstract

**Objective:**

Morular metaplasia (MM) is a benign epithelial metaplasia that sometimes appears in atypical endometrial hyperplasia (AEH) and endometrioid endometrial carcinoma (EEC). However, the clinical implications of MM for fertility-preserving treatment in AEH and EEC patients are unclear. This study investigated the clinical features and impact of MM on the efficacy of fertility-preserving treatment.

**Methods:**

We retrospectively studied 427 AEH and EEC patients who received fertility-preserving treatment. Clinical features, treatment efficacy, and onco-fertility results were compared between patients with and without MM.

**Results:**

MM appeared in 147 of 427 (34.4%) patients. Among them, 49 (33.3%) had MM only before treatment (BEF group), 32 (21.8%) had sustained MM before and during treatment (SUS group), and 66 (44.9%) had MM only during treatment (DUR group). The BEF group had a higher 12-month CR rate (98.0% vs 85.7%, *p* = 0.017) and shorter therapeutic duration to achieve CR (4.0 vs 5.7 months, *p* = 0.013) than the non-MM group had. In comparison with the non-MM group, the SUS and DUR groups had a lower CR rate after 7 months of treatment (SUS vs non-MM, 37.5% vs 61.1%, *p* = 0.010; DUR vs non-MM 33.3% vs. 61.1%, *p* < 0.001), and a longer median therapeutic duration to achieve CR (SUS vs non-MM, 7.6 vs. 4.0 months, *p* = 0.037; DUR vs non-MM, 7.9 vs. 4.0 months, *p* < 0.001).

**Conclusion:**

Appearance of MM only before treatment was positively correlated with outcome of fertility-preserving treatment, while sustained MM or appearance of MM only during treatment implied poorer outcome of fertility-preserving treatment in AEH and EEC patients.

**Supplementary Information:**

The online version contains supplementary material available at 10.1007/s00404-021-06382-3.

## Introduction

Morular metaplasia (MM) is a benign epithelial metaplasia [1–3] that arises from immature squamous epithelial cell differentiation [4, 5]. As a rare condition of epithelial metaplasia, MM presents as morphologically rounded, well-circumscribed aggregations of uniform, oval, or spindle-shaped cell clusters [6, 7]. MM might sometimes appear in the endometrium and is almost always associated with focal, complex endometrial glandular lesions [1, 8]. Studies have shown that MM in endometrial lesions is associated with benign diseases or relatively inert malignancy [3, 6, 8, 9]. Although the mechanisms of occurrence of MM in the endometrium are not sufficiently clear, it has been reported that the occurrence of MM is associated with chronic endometritis, submucosal leiomyoma, irradiation, exogenous hormone therapy, and intrauterine devices [1, 2].

MM is also found in atypical endometrial hyperplasia (AEH) or early-stage endometrioid endometrial cancer (EEC) lesions receiving fertility-preserving treatment. This pathological phenomenon appears either before or during progestin-based treatment in endometrial lesions. However, the clinical implications of the appearance of MM are not clear in these patients receiving fertility-preserving treatment.

In this study, we retrospectively analyzed AEH and EEC patients who received fertility-preserving treatment at the Obstetrics and Gynecology Hospital of Fudan University. Clinical characteristics and treatment outcomes of patients with MM before and/or during fertility-preserving treatment were analyzed and compared with those in patients without MM. Appearance of MM in endometrial lesions only before fertility-preserving treatment was associated with higher complete response (CR) rate and shorter treatment duration to achieve CR in AEH and EEC patients compared with those without MM.

## Materials and methods

### Study population

**Fig. 1:**
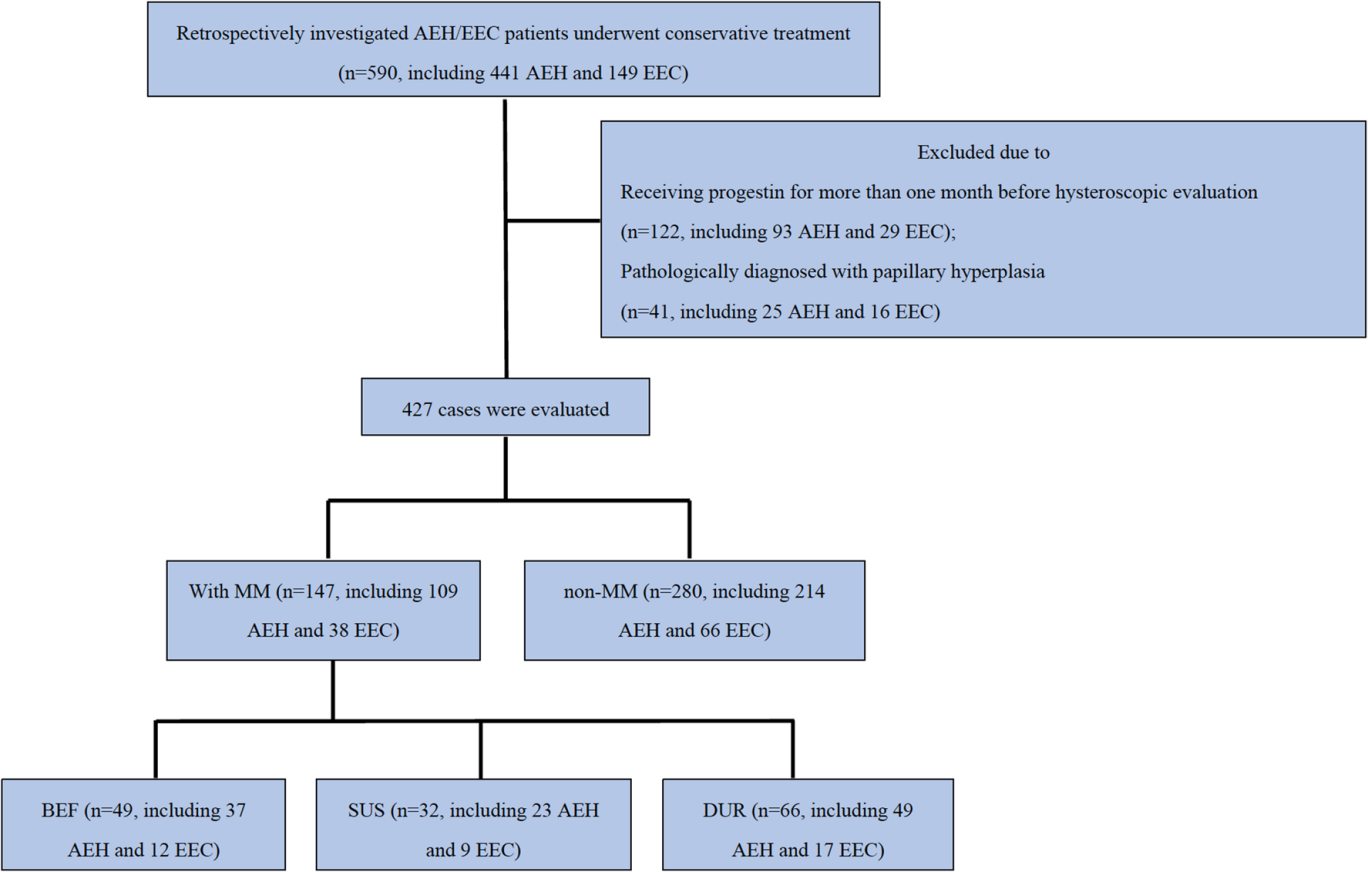
Flowchart of our retrospective study design. *AEH* atypical endometrial hyperplasia, *EEC* endometrioid endometrial carcinoma, *MM* morular metaplasia, *BEF* MM before treatment group, *SUS* sustained MM group, *DUR* MM during treatment group

This was a retrospective study of 590 consecutive patients (441 AEH and 149 EEC) who received fertility-preserving treatment at the Obstetrics and Gynecology Hospital of Fudan University between March 2013 and October 2019 (Fig. 1). Because it has been reported that the appearance of MM is correlated with hormone use, we excluded 122 patients who received progestin treatment for > 1 month before the first endometrial evaluation at our hospital. Another 41 patients with papillary hyperplasia in endometrial lesions were also excluded because this pathological type might also have some impact on fertility-preserving treatment. Ultimately, 427 patients (323 AEH and 104 EEC grade 1) were included. All the patients received standardized evaluation and treatment, and patient information was prospectively collected and recorded during treatment and follow-up. The study was approved by the Ethics Committee of Obstetrics and Gynecology Hospital of Fudan University.

The inclusion and exclusion criteria for fertility-sparing treatment followed National Comprehensive Cancer Network guidelines [10] and included: (1) histologically proven AEH, or well-differentiated EEC grade 1 without myometrial invasion; (2) no signs of suspicious extrauterine involvement on enhanced magnetic resonance imaging (MRI), enhanced computed tomography (CT), or ultrasound; (3) age < 45 years; (4) strong willingness to preserve fertility; (5) no contraindication for progestin treatment or pregnancy; (6) not pregnant; and (7) good compliance for treatment. Written informed consent was obtained from all patients before initiating treatment. Patients with MM in endometrial lesions before and/or during fertility-preserving treatment were included in the study group. Patients with AEH and/or EEC grade 1 only were included as the non-MM group.

### Pathological diagnosis

All patients were pathologically diagnosed through dilation and curettage with or without hysteroscopy. Pathological diagnosis was confirmed by two experienced gynecological pathologists according to the World Health Organization pathological classifications (2014). If their opinions differed, a seminar was held in the Pathology Department to achieve a final diagnosis. We performed another hysteroscopy within 1 month after initial pathological diagnosis if the patient was diagnosed by dilation and curettage without hysteroscopy.

Diagnosis of morular metaplasia was based on Blaustein’s Pathology of the Female Genital Tract. Briefly, MM is composed of rounded aggregates or syncytial sheets of cells that often fill the glandular lumina. The constituent cells have central bland, round, ovoid, or spindle shapes, evenly spaced nuclei, and sometimes show small nucleoli [2].

### Fertility-preserving treatment and evaluation of treatment outcomes

Fertility-preserving treatment was initiated as soon as comprehensive evaluation was completed, and the multidisciplinary team judged the patient suitable for treatment. Therapeutic regimens were decided by physicians. Most patients received oral megestrol acetate (MA) at 160 mg/day with or without metformin (500 mg, three times daily). The other patients were treated with a levonorgestrel-releasing intrauterine system (LNG-IUS), ethinylestradiol cyproterone (Diane-35), or gonadotropin-releasing hormone analog (GnRH-a) plus letrozole. A comprehensive hysteroscopic evaluation was performed every 3 months during treatment to evaluate therapeutic efficacy [11]. Endometrial lesions were removed under hysteroscopy, and an endometrial biopsy was randomly performed if no obvious lesion was found.

Treatment response was evaluated histologically using specimens obtained during each hysteroscopy. Complete response (CR) was defined as no hyperplasia or cancerous lesion. Partial response (PR) was defined as pathological improvement. Stable disease (SD) was defined as persistence of the initially diagnosed lesion. Progressive disease (PD) was defined as evidence of EC in patients with AEH, or evidence of more severe pathological findings, myometrial invasion, or extrauterine metastasis in EEC patients.

Once the patient achieved CR, the same regimen was continued for another 2–3 months for consolidation. Hysteroscopy was performed 3 months after the first CR, for confirmation. The therapeutic duration to achieve CR was calculated from the time of initiating treatment to the time of first pathological CR diagnosis, if no hyperplasia or cancerous lesion was found in two consecutive hysteroscopic evaluations. All patients desiring fertility were encouraged to receive assisted reproductive treatments such as in vitro fertilization after CR. Low-dose progestin, oral contraceptive pills, or LNG-IUS was used to prevent recurrence in patients who did not have a recent parental plan. Hysterectomy was strongly recommended for patients who had SD for 6 months, PR for 9 months, or PD at any time during treatment. For patients who refused hysterectomy, alternative treatments including Diane-35 (one pill/day for 21 days of a 28-day cycle) combined with metformin (500 mg, three times daily), LNG-IUS insertion, or intramuscular injection of GnRH-a were given according to the recommendations of a multidisciplinary team.

Patients were followed up every 3–6 months after CR. Ultrasound evaluation was made at each follow-up visit and endometrial biopsy using Pipelle was performed every 6 months during follow-up. Patients were followed up until October 2020. The median follow-up time, from initial treatment in our center to the last follow-up, was 26.2 months (range 3.2–90.3 months). The median follow-up time from the date of achieving CR to the last follow-up was 24.3 months (range 3.1–76.9 months).

### Data collection

General information about the patients, including age, weight, height, basic blood pressure, and comorbidities (e.g., hypertension or diabetes) was collected before any treatment was given. Blood samples before initiating fertility-preserving treatment were collected and tested for fasting blood glucose, fasting insulin, and lipid profiles. All blood samples were collected and examined in the laboratory of the Obstetrics and Gynecology Hospital as previously described [12]. Body mass index (BMI) was calculated as weight (kg)/height^2^ (m^2^). BMI ≥ 25 kg/m^2^ was considered as overweight [13]. Insulin resistance (IR) was estimated using the homeostasis model assessment of insulin resistance (HOMA-IR). The HOMA-IR index was calculated as fasting blood glucose (mmol/L) × fasting insulin (μU/mL)/22.5 [14]. When HOMA-IR was ≥ 2.96, we defined the patient as having IR [15]. The diagnostic criteria for metabolic syndrome (MS) have been described previously [12]. Symptoms of chronic estrogen stimulation also were collected, including menometrorrhagia, prolonged menstruation, irregular menses, early menarche, amenorrhea, polycystic ovary syndrome (PCOS). When the time of menarche was 11 years old or less, we defined the patient as early menarche [16]. Diagnosis of PCOS was based on Rotterdam Consensus Criteria [17].

### Statistical analysis

All descriptive data are presented as mean and SD for data with a Gaussian distribution and as median plus range for non-Gaussian distributed data. The categorical variables are presented as frequency and percentage. Numerical variables were analyzed using Student’s *t *test or Mann–Whitney *U* test. The chi-square test was used to analyze categorical variables, except if the expected frequency was < 5; when this was the case, Fisher’s exact test or likelihood-ratio chi-square test was used. Therapeutic duration was estimated by the Kaplan–Meier method and compared between groups using the log-rank test. A Cox regression model was used for univariate and multivariate analyses of the relationship between covariates and CR during fertility-preserving treatment. A logistic regression model was used to measure the association between morular metaplasia and risk factors in multivariate analyses. SPSS version 23.0 (IBM, Armonk, NY, USA) was used for all statistical analyses, and two-tailed *p *values < 0.05 were considered statistically significant.

## Results

### General characteristics of patients with or without MM

Totally, twenty-four patients (24/427, 5.6%) were lost to follow-up till October 2020, including 12 patients who cannot be contacted, 4 patients who did not come back to our clinic after being contacted and 10 patients who rejected to be followed up. Among those patients, 7 (7/147, 4.8%) were in MM group and 17 (17/280, 6.1%) were in non-MM group.

**Fig. 2:**
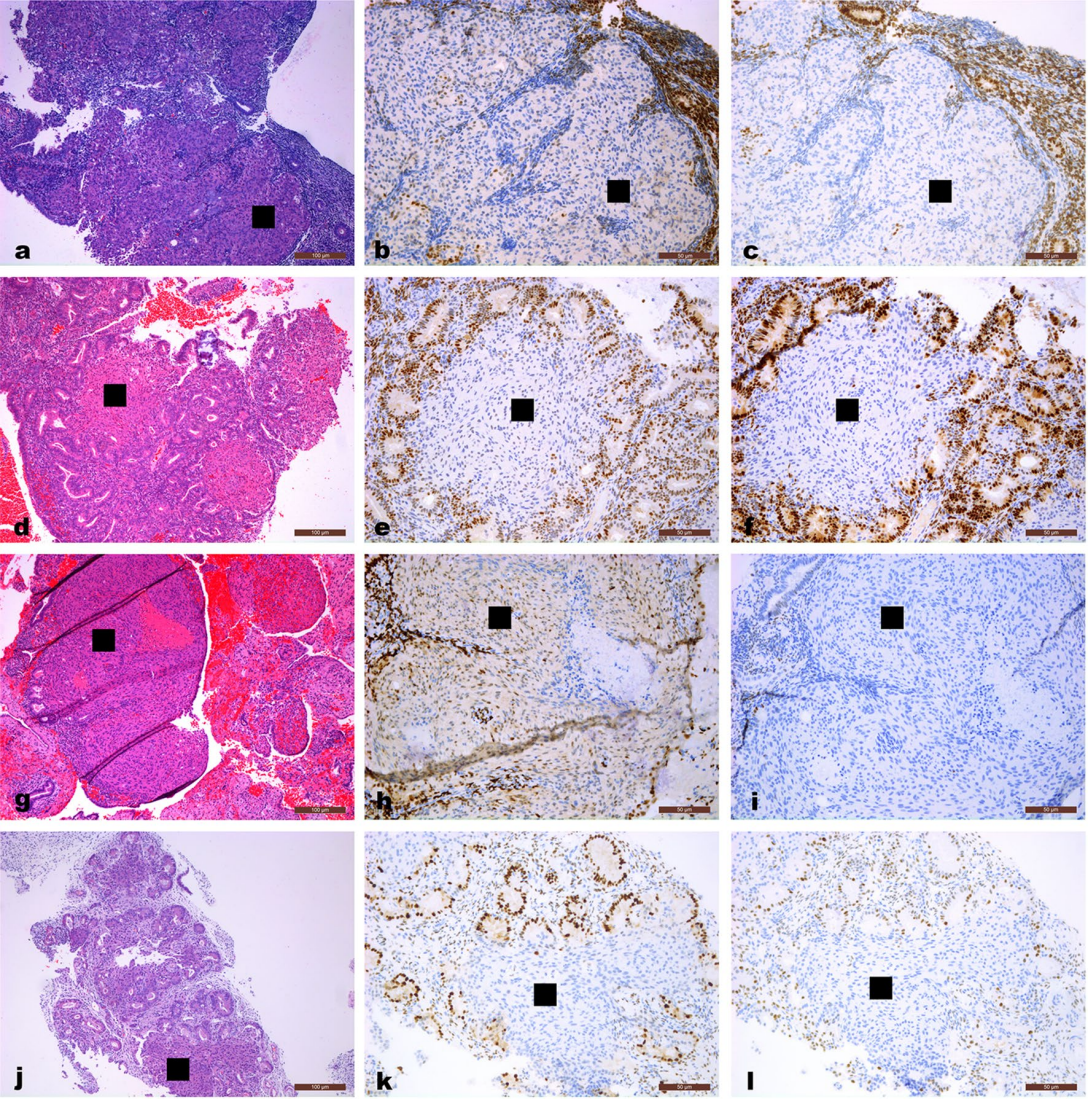
Morular metaplasia (indicated by the square) in endometrial lesions and its immuno-histochemical staining for estimation of ER (**b**, **e**, **h**,** k**) and PR (**c**, **f**, **i**, **l**). Expression of PR was all negative among morular metaplasia. **a**–**c** Morular metaplasia appeared only before progestin treatment. **d**–**i** Sustained morular metaplasia. (**d**–**f**, before progestin treatment; **g**–**i**, during progestin treatment). **j**–**l** Morular metaplasia appeared only during progestin treatment. (**a**, **d**, **g**, **j**: original magnification × 10; other photographs: magnification × 20). ER, estrogen receptor; PR, progesterone receptor

MM appeared in 147 (34.4%) patients. Among them, 49 (33.3%) had MM only before treatment that disappeared after initiation of treatment (BEF group); 32 (21.8%) had sustained MM before and during treatment (SUS group); and 66 (44.9%) had MM only during treatment (DUR group). The remaining 280 (65.6%) patients comprised the non-MM group who had only AEH or EEC in endometrial lesions, without any other specific pathological phenomena. In the DUR group, median duration from initiation of treatment to the appearance of MM was 4.2 months (range 1.0–21.4 months). Representative images of MM are shown in Fig. 2. Expression of progestin receptors in MM in endometrial lesions was all negative.

General characteristics of patients with and without MM are presented in Table 1. Patients in the SUS group (age 29.0 years, *p* = 0.001) and DUR group (age 31.0 years, *p* = 0.022) were both younger than those in the non-MM group (age 33.0 years). Follicle-stimulating hormone (FSH) in the BEF group was higher than in the non-MM group (6.87 vs 6.27 mIU/mL, *p* = 0.048). There were no other significant differences between the SUS and DUR groups and non-MM group. More patients in the DUR group had metabolic disorders than those in the BEF group, which was demonstrated by higher BMI (25.80 vs 23.01 kg/m^2^, *p* = 0.003), and more patients with MS (54.5% vs 32.7%, *p* = 0.020) and hypertension (28.8% vs 12.2%, *p* = 0.033).

**Table 1:** General characteristics of the study population

	Comparison between MM groups and non-MM group							Comparison among MM groups		
	Non-MM	BEF	*p*-value^a^	SUS	*p*-value^b^	DUR	*p*-value^c^	BEF versus SUS	BEF versus DUR	SUS versus DUR
Patient number, *n* (%)	280 (65.6%)	49 (11.5%)	–	32 (7.5%)	–	66 (15.4%)	–	–	–	–
Age at diagnosis (years) Median (range)	33.0 (20.0–43.0)	32.0 (18.0–44.0)	0.177	29.0 (24.0–40.0)	**0.001**	31.0 (25.0–44.0)	**0.022**	0.093	0.687	0.064
BMI (kg/m^2^) Median (range)	24.34 (10.66–51.56)	23.01 (15.94–41.52)	0.130	23.44 (16.38–32.65)	0.084	25.80 (16.80–43.55)	0.013	0.703	**0.003**	0.004
DM, *n* (%)	27 (9.6%)	2 (4.1%)	0.320	0 (0.0%)	0.132	6 (9.1%)	0.891	0.671	0.501	0.190
Hypertension, *n* (%)	63 (22.5%)	6 (12.2%)	0.104	5 (15.6%)	0.372	19 (28.8%)	0.280	0.918	**0.033**	0.155
IR, *n* (%)	105 (37.5%)	16 (32.7%)	0.516	9 (28.1%)	0.297	33 (50.0%)	0.062	0.666	0.063	**0.040**
MS, *n* (%)	132 (47.1%)	16 (32.7%)	0.060	9 (28.1%)	**0.041**	36 (54.5%)	0.279	0.666	**0.020**	**0.014**
*E*2 (pg/ml) Median (range)	55 (4–1237)	55 (12–392)	0.294	54 (8–270)	0.896	52 (2–583)	0.434	0.440	0.773	0.666
*T* (ng/ml) Median (range)	0.42 (0.01–2.15)	0.37 (0.02–1.13)	0.582	0.44 (0.01–1.55)	0.273	0.35 (0.01–23.56)	0.391	0.175	0.801	0.130
FSH (mIU/ml) Median (range)	6.27 (0.56–49.18)	6.87 (1.43–26.67)	**0.048**	6.21 (1.52–11.87)	0.648	5.87 (0.94–53.00)	0.923	0.267	0.086	0.570
LH (mIU/ml) Median (range)	5.30 (0.10–389.00)	4.33 (0.20–76.39)	0.912	6.73 (0.22–50.87)	0.262	4.49 (0.53–17.55)	0.606	0.444	0.741	0.160
SHBG (nmol/L) Median (range)	30.25 (3.50–200.00)	33.50 (13.20–187.70)	0.152	31.20 (9.00–109.70)	0.966	26.45 (6.06–200.00)	0.437	0.271	0.071	0.560
Treatment, *n* (%)			0.967		0.579		0.052	0.587	0.099	0.479
MA	154 (55.0%)	26 (53.1%)		20 (62.5%)		38 (57.6%)		–	–	–
MA + MET	83 (29.6%)	15 (30.6%)		9 (28.1%)		25 (37.9%)		–	–	–
Other^d^	43 (15.4%)	8 (16.3%)		3 (9.4%)		3 (4.5%)		–	–	–
Prevention method			0.100		0.138		0.673	0.261	0.607	0.403
LNG-IUS	93 (52.5%)	14 (43.8%)		7 (38.9%)		20 (52.6%)		–	–	–
MPA	24 (13.6%)	1 (3.1%)		2 (11.1%)		3 (7.9%)		–	–	–
OC	48 (27.1%)	14 (43.8%)		9 (50.0%)		13 (34.2%)		–	–	–
None	12 (6.8%)	3 (9.4%)		0 (0.0%)		2 (5.3%)		–	–	–
Method for pregnancy^e^, *n* (%)			0.520		0.139		0.915	0.448	0.871	0.149
Natural pregnancy	21 (14.2%)	2 (6.3%)		0 (0.0%)		4 (11.4%)		–	–	–
Ovulation induction	31 (20.9%)	9 (28.1%)		9 (34.7%)		8 (22.9%)		–	–	–
IVF	79 (53.4%)	18 (56.3%)		14 (53.8%)		20 (57.1%)		–	–	–
Unknown	17 (11.5%)	3 (9.4%)		3 (11.5%)		3 (8.6%)		–	–	–

*MM* morular metaplasia, *BEF* MM before treatment group, *SUS* sustained MM group, *DUR* MM during treatment group, *BMI* body mass index, BMI = weight/height^2^, *DM* diabetes mellitus, *IR* insulin resistance, *MS* metabolic syndrome, *E2* estradiol, *T* testosterone, *FSH* follicle-stimulating hormone, *LH* luteinizing hormone, *SHBG* sex hormone binding globulin, *MA* megestrol acetate, 160 mg/day, *MET* metformin, 1500 mg/day, *LNG-IUS* levonorgestrel-releasing intrauterine system, *MPA* medroxyprogesterone acetate, *OC* oral contraceptive pills, *IVF* in vitro fertilization

^a^*p*-value: difference between BEF and non-MM group

^b^*p*-value: difference between SUS and non-MM group

^c^*p*-value: difference between DUR and non-MM group

^d^Other regimen: levonorgestrel-releasing intrauterine system (LNG-IUS), ethinylestradiol cyproterone (Diane-35), or gonadotropin-releasing hormone analog (GnRH-a) plus letrozole

^e^Among patients who plan for parenthood

370 patients received MA with or without metformin while 57 patients received other regimens including 54 patients receiving LNG-IUS, 1 patient receiving Diane-35 and 2 patients receiving GnRH-a plus letrozole. The reason of using Diane-35 on the only one patient was her typical symptoms of PCOS and starting Diane-35 for less than 1 month before coming to our center. One patient received GnRH-a plus letrozole because of her high risk of thrombosis and hepatic dysfunction. The other because of her thrombophlebitis of left forearm.

No significant differences were found in treatment regimens, disease prevention methods, preparation for pregnancy, or conservative treatment regimens among these groups.

### Implications of MM for fertility-preserving treatment outcome

**Fig. 3:**
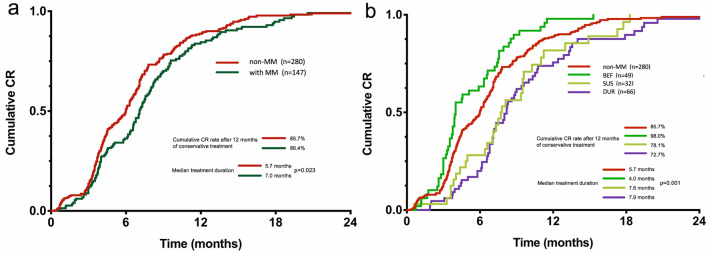
Cumulative CR rate in AEH and EEC patients. **a** Cumulative CR rate in patients with and without morular metaplasia. **b** Cumulative CR rate among three subgroups of patients with morular metaplasia and without morular metaplasia. *CR* complete response, *MM* morular metaplasia, *AEH* atypical endometrial hyperplasia, *EEC* endometrioid endometrial carcinoma, *BEF* MM before treatment group, *SUS* sustained MM group, *DUR* MM during treatment group

To investigate the clinical implications of MM for fertility-preserving treatment in AEH and EEC patients, we analyzed the CR rate and therapeutic duration to achieve CR in patients with or without MM (Table 2, Fig. 3a, b). The BEF group had better conservative treatment outcome compared with the non-MM group, while the SUS and DUR groups had worse treatment outcome compared with the non-MM group. The BEF group had a significantly higher cumulative CR rate after 10 months of treatment compared with the non-MM group (91.8% vs. 80.0%, *p* = 0.048). There was a similar tendency in cumulative CR rate after 12 months of treatment (98.0% vs. 85.7%, *p* < 0.017). The median therapeutic duration to achieve CR in the BEF group was significantly shorter than in the non-MM group (4.0 vs 5.7 months, *p* = 0.013). In contrast, in comparison with the non-MM group, the SUS and DUR groups had a lower CR rate after 7 months of treatment (SUS vs non-MM, 37.5% vs 61.1%, *p* = 0.010; DUR vs non-MM, 33.3% vs 61.1%, *p* < 0.001). The SUS and DUR groups both had a longer median therapeutic duration to achieve CR than the non-MM group had (SUS vs non-MM, 7.6 vs 4.0 months, *p* = 0.037; DUR vs non-MM, 7.9 vs 4.0 months, *p* < 0.001).

**Table 2:** The outcome of conservative treatment

	Comparison between MM groups and non-MM group							Comparison among MM groups		
	Non-MM	BEF	*p*-value^a^	SUS	*p*-value^b^	DUR	*p*-value^c^	BEF versus SUS	BEF versus DUR	SUS versus DUR
Median follow-up duration (range) (months)	27.9 (3.2–90.3)	26.8 (6.2–69.3)	0.703	23.4 (6.0–79.3)	0.309	23.8 (7.7–67.6)	0.446	0.288	0.403	0.682
Median treatment duration to CR (range) (months)	5.7 (0.3–28.2)	4.0 (0.7–15.3)	**0.013**	7.6 (0.6–18.3)	**0.037**	7.9 (1.9–30.4)	**< 0.001**	**< 0.001**	**< 0.001**	0.388
CR^d^, *n* (%)										
7 months CR	171/280 (61.1%)	35/49 (71.4%)	0.167	12/32 (37.5%)	**0.010**	22/66 (33.3%)	**< 0.001**	**0.002**	**< 0.001**	0.684
10 months CR	224/280 (80.0%)	45/49 (91.8%)	**0.048**	22/32 (68.8%)	0.140	43/66 (65.2%)	**0.010**	**0.007**	**0.001**	0.724
12 months CR	240/280 (85.7%)	48/49 (98.0%)	**0.017**	25/32 (78.1%)	0.256	48/66 (72.7%)	**0.011**	**0.011**	**< 0.001**	0.565
Pregnancy^e^, *n* (%)	52/148 (35.1%)	12/32 (37.5%)	0.800	7/26 (26.9%)	0.415	9/35 (25.7%)	0.288	0.393	0.299	0.915
Live-birth^e^, *n* (%)	35/148 (23.6%)	9/32 (28.1%)	0.593	5/26 (19.2%)	0.621	7/35 (20.0%)	0.644	0.431	0.436	0.940
Median duration to relapse (range) (months)	13.1 (2.5–58.8)	10.3 (7.1–37.8)	0.988	17.1 (4.9–25.2)	0.224	16.0 (7.2–28.2)	0.464	0.534	0.313	0.740
Relapse^f^, *n* (%)	28/262 (10.7%)	4/49 (8.2%)	0.594	6/30 (20.0%)	0.228	8/61 (13.1%)	0.587	0.235	0.408	0.585

*MM* morular metaplasia, *BEF* MM before treatment group, *SUS* sustained MM group, *DUR* MM during treatment group, *CR* complete response

^a^*p*-value: difference between BEF and non-MM group

^b^*p*-value: difference between SUS and non-MM group

^c^*p*-value: difference between DUR and non-MM group

^d^Cumulative CR rate after 12 months of conservative treatment

^e^Among patients who plan for parenthood

^f^Cumulative relapse rate in 24 months after CR

The pregnancy and relapse rates are shown in Table 2. Among the 402 patients who achieved CR, 241 planned for parenthood. At the time of last follow-up, 80/241 (33.2%) patients achieved at least one pregnancy, including 52/148 (35.1%) patients without and 28/93 (30.1%) with MM. The live birth rate was 23.6% (35/148 cases) and 22.6% (21/93 cases) among patients without or with MM, respectively. No significant differences were found among the groups with or without MM in terms of pregnancy and live birth rates.

Among the 402 patients who achieved CR, 46 (11.4%) relapsed within 24 months after CR, including 28/262 (10.7%) without and 18/140 (12.9%) with MM. The median interval from CR to recurrence was 13.1 months (range 2.5–58.8 months) for patients without and 14.0 months (range 4.9–37.8 months) for patients with MM. No significant differences in relapse were found among the groups with or without MM.

### Factors associated with CR

**Fig. 4:**
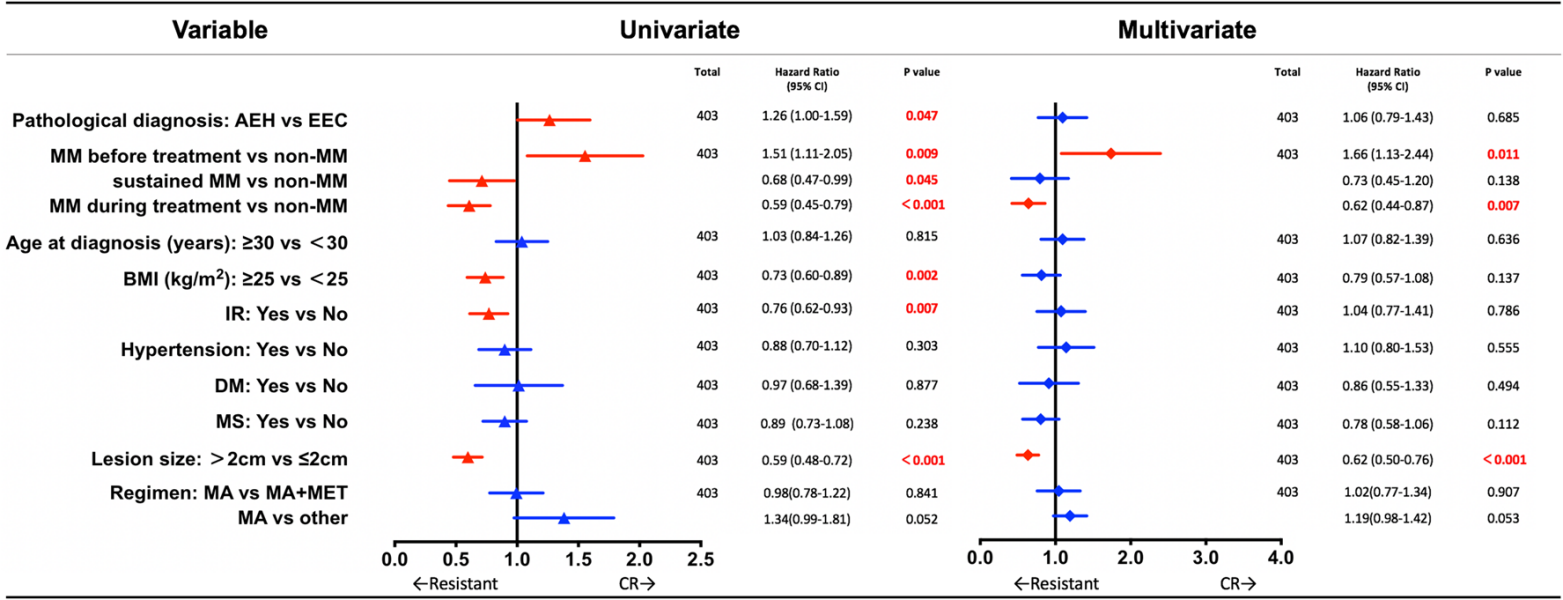
Uni- and multi-variate analyses for factors associated with complete response according to Cox regression model. *95% CI* 95% confidence interval, *AEH* atypical endometrial hyperplasia, *EEC* endometrioid endometrial carcinoma, *MM* morular metaplasia, *BMI* body mass index, *IR* insulin resistance, *DM* diabetes mellitus, *MS* metabolic syndrome, *CR* complete response

We performed univariate and multivariate analyses to determine factors related to CR in AEH and EEC patients receiving fertility-preserving treatment (Fig. 4). Univariate Cox regression analysis showed that MM during treatment [hazard ratio (HR) 0.68, 95% confidence interval (CI) 0.47–0.79, *p* = 0.045], sustained MM (HR 0.59, 95% CI 0.45–0.79, *p* < 0.001), BMI ≥ 25 kg/m^2^ (HR 0.73, 95% CI 0.60–0.89, *p* = 0.002), IR (HR 0.76, 95% CI 0.62–0.93, *p* = 0.007), and lesion size > 2 cm (HR 0.59, 95% CI 0.48–0.72, *p* < 0.001) were correlated with lower CR rate. In contrast, AEH (HR 1.26, 95% CI 1.0–1.59, *p* = 0.047) and MM before treatment (HR 1.51, 95% CI 1.11–2.05, *p* = 0.009) were correlated with higher CR rate.

Multivariate analyses showed that MM before treatment (HR 1.66, 95% CI 1.13–2.44, *p* = 0.011) was correlated with higher CR rate after adjusting for pathological diagnosis, MM, age at diagnosis, BMI, IR, hypertension, DM, MS, lesion size and regimen. MM during treatment (HR 0.62, 95% CI 0.44–0.87, *p* = 0.007) and lesion size > 2 cm (HR 0.62, 95% CI 0.50–0.76, *p* < 0.001) were independent risk factors for lower CR rate.

### Factors associated with appearance of MM

**Fig. 5:**
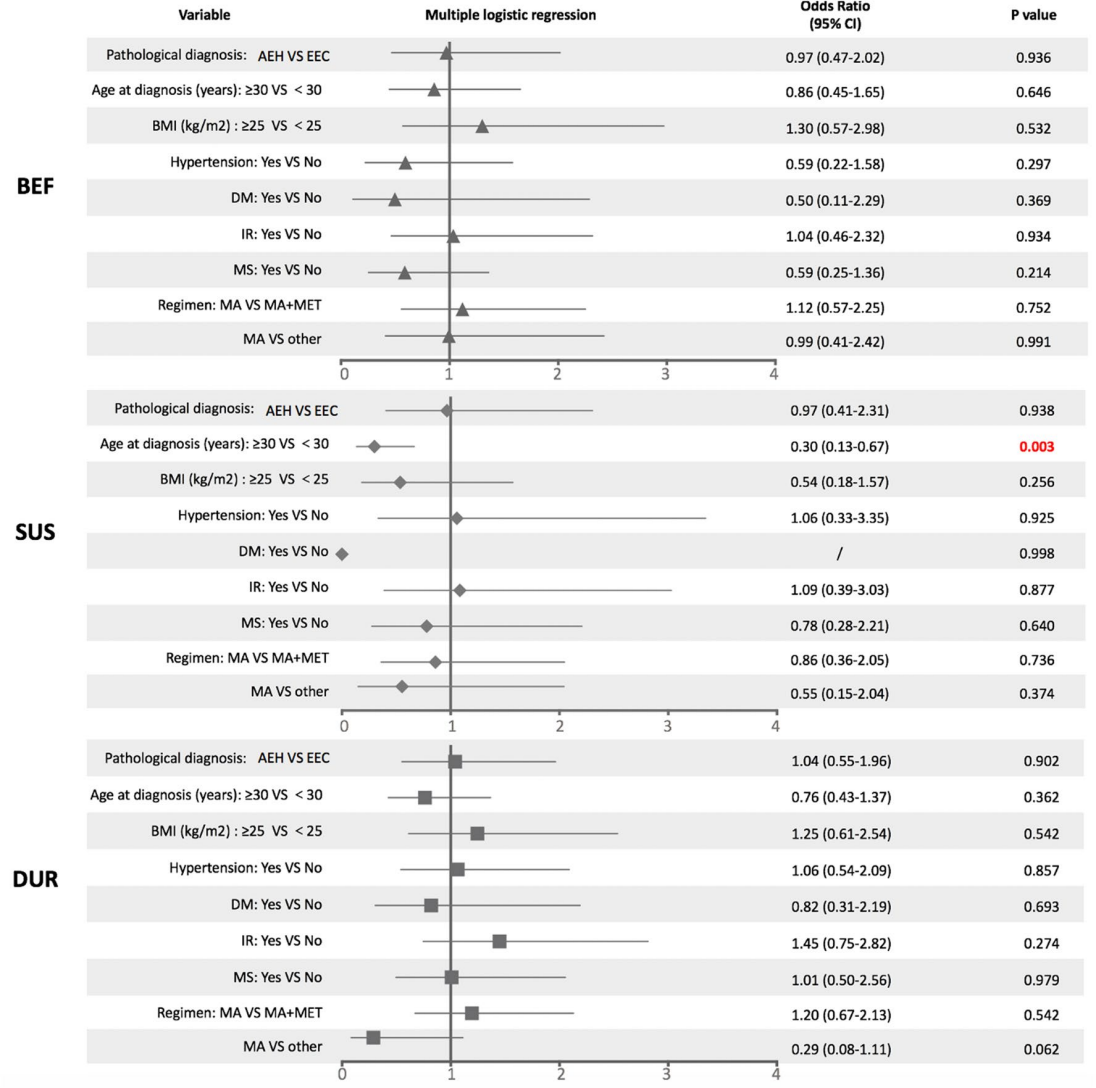
Multi-variate subgroup analyses for factors associated with morular metaplasia according to logistic regression. *BEF* MM before treatment group, *SUS* sustained MM group, *DUR* MM during treatment group, *95% CI* 95% confidence interval, *AEH* atypical endometrial hyperplasia, *EEC* endometrioid endometrial carcinoma, *BMI* body mass index, *DM* diabetes mellitus, *IR* insulin resistance, *MS* metabolic syndrome, *MA* megestrol acetate, *MET* metformin, *other* including levonorgestrel-releasing intrauterine system (LNG-IUS), ethinylestradiol cyproterone (Diane-35), or gonadotropin-releasing hormone analog (GnRH-a)

To explore the possible risk factors associated with the appearance of MM in endometrial lesions, we first compared symptoms of chronic estrogen stimulation between MM group and non-MM group in Online Resource 1 and the results showed no significant difference. Second, logistic regression analysis was carried out in the three MM subgroups (Fig. 5). Multivariate logistic regression analysis showed that patients older than 30 years were less likely to have sustained MM (odds ratio 0.30, 95% CI 0.13–0.67, *p* = 0.003) after adjusting for pathological diagnosis, age at diagnosis, BMI, hypertension, DM, IR, MS and treatment regimens. No other risk factors were found to be correlated with appearance of MM either before or during fertility-preserving treatment.

## Discussion

In this study, we retrospectively analyzed the impact of MM on the outcome of fertility-preserving treatment in AEH and EEC patients. Our study showed that appearance of MM in different periods of progestin treatment had different clinical implications. Compared with the non-MM group, the BEF group was correlated with a higher CR rate and shorter therapeutic duration to achieve CR, while the DUR and SUS groups had poorer fertility-preserving treatment outcome.

The etiology of MM is not well understood. We did not find obvious risk factors correlated with the appearance of MM. Previous studies have shown that MM might be caused by chronic inflammation, such as chronic endometritis, submucosal leiomyoma, irradiation, or intrauterine devices [6, 7]. Other studies have reported that MM is associated with unopposed estrogen exposure or progestin treatment [18, 19]. Although the BEF, DUR and SUS groups had the same pathological characteristics, the different fertility-preserving treatment results implied that the etiology of MM in the BEF group differed from that in the DUR and SUS groups. MM that appeared only before treatment might be associated with prolonged estrogen exposure and thus sensitivity to progestin treatment. The SUS and DUR groups had similar fertility-preserving treatment results, which indicated similar etiology of MM in these two groups. The fact that more patients in the DUR group suffered from metabolic disorders such as hypertension, higher BMI and MS indicated that chronic inflammation caused by metabolic disorders might induce the appearance of MM, which could explain the poorer fertility-preserving treatment outcome in these two groups. It has been widely reported that chronic inflammation induced by metabolic disorders is negatively correlated with fertility-preserving treatment in AEH and EEC patients [12, 20, 21]. Further studies that test this hypothesis are warranted.

Our study had some clinical implications for fertility-preserving treatment in AEH and EEC patients. Appearance of MM before fertility-preserving treatment and its disappearance after treatment initiation implied a better treatment outcome, while sustained MM or appearance of MM only during fertility-preserving treatment implied poorer treatment results. These patients should be paid more attention, and treatment of complicated metabolic disorders might help improve fertility-preserving treatment. Furthermore, the fact that the SUS and DUR groups were younger than the non-MM group suggested that young AEH and EEC patients with metabolic disorders were more likely to have MM and develop resistance to progestin treatment.

The retrospective nature of our study was its main limitation and this study is subject to selection bias. A prospective study of both AEH and EEC patients might provide more information about the impact of MM on the result of fertility-preserving treatment in these patients. Additionally, molecular classification of endometrial lesion might help investigate the mechanism of appearance of MM. At last, there may be a small possibility that the morular metaplasia was not identified at the point of diagnosis.

In conclusion, our study showed that MM that appeared only before progestin treatment was positively correlated with the efficacy of fertility-preserving treatment in AEH and EEC patients, with a higher CR rate and shorter therapeutic duration to achieve CR. Patients with sustained MM or appearance of MM only during progestin treatment had poorer outcome of fertility-preserving treatment compared with patients in the non-MM group. Further studies are needed to confirm our findings and investigate the mechanisms involved.

## Supplementary Information

Below is the link to the electronic supplementary material.

Supplementary file1 (DOCX 14 KB)
