# Supplementary material for: Clinical implications of morular metaplasia in fertility-preserving treatment for atypical endometrial hyperplasia and early endometrial carcinoma patients
Source: Arch Gynecol Obstet. 2022 Mar 4;306(4):1135–46. doi: 10.1007/s00404-021-06382-3 (PMC9470654; doi:10.1007/s00404-021-06382-3)
Supplement: Supplementary file 1 — Supplementary file1 (DOCX 14 KB) [file 404_2021_6382_MOESM1_ESM.docx]

|  | non-MM | MM | p-value |
| --- | --- | --- | --- |
| Menometrorrhagia, n (%) | 44(15.7%) | 24(16.3%) | 0.870 |
| Prolonged menstruation, n (%) | 27(9.6%) | 9(6.1%) | 0.214 |
| Irregular menses, n (%) | 184(65.7%) | 95(64.6%) | 0.822 |
| Early menarche, n (%) | 11(3.9%) | 2(1.4%) | 0.234 |
| Amenorrhea, n (%) | 5(1.8%) | 2(1.4%) | 1.000 |
| PCOS, n (%) | 70(25.0%) | 49(33.3%) | 0.068 |

**Online Resource 1 (Supplementary Table 1)**. Symptoms of chronic estrogen stimulation

Footnote：

MM, morular metaplasia; PCOS, polycystic ovary syndrome.
